# Advancing Cardiovascular Drug Screening Using Human Pluripotent Stem Cell-Derived Cardiomyocytes

**DOI:** 10.3390/ijms25147971

**Published:** 2024-07-21

**Authors:** Jisun Oh, Oh-Bin Kwon, Sang-Wook Park, Jun-Woo Kim, Heejin Lee, Young-Kyu Kim, Eun Ji Choi, Haiyoung Jung, Dong Kyu Choi, Bae Jun Oh, Sang-Hyun Min

**Affiliations:** 1 New Drug Development Center, Daegu-Gyeongbuk Medical Innovation Foundation (K-MEDI Hub), Daegu 41061, Republic of Korea; joh@kmedihub.re.kr (J.O.); kob325@kmedihub.re.kr (O.-B.K.); margarte@hanmail.net (J.-W.K.); leeheejin@kmedihub.re.kr (H.L.); kimyk0114@kmedihub.re.kr (Y.-K.K.); 2 Department of Oral Biochemistry, School of Dentistry, Chonnam National University, Gwangju 61186, Republic of Korea; swpark@chonnam.ac.kr; 3 Aging Convergence Research Center, Korea Research Institute of Bioscience and Biotechnology (KRIBB), Daejeon 34141, Republic of Korea; cej2017@kribb.re.kr (E.J.C.); haiyoung@kribb.re.kr (H.J.); 4 Immunotherapy Research Center, Korea Research Institute of Bioscience and Biotechnology (KRIBB), Daejeon 34141, Republic of Korea; 5 Department of Functional Genomics, Korea University of Science and Technology (UST), Daejeon 34113, Republic of Korea; 6 School of Life Science and Biotechnology, BK21 FOUR KNU Creative BioResearch Group, Kyungpook National University, Daegu 41566, Republic of Korea; dongkyu@knu.ac.kr; 7 Department of Innovative Pharmaceutical Sciences, Kyungpook National University, Daegu 41566, Republic of Korea

**Keywords:** human pluripotent stem cells, cardiomyocytes, drug screening, cardiovascular pharmacology, high-throughput assays

## Abstract

Human pluripotent stem cell-derived cardiomyocytes (hPSC-CMs) have emerged as a promising tool for studying cardiac physiology and drug responses. However, their use is largely limited by an immature phenotype and lack of high-throughput analytical methodology. In this study, we developed a high-throughput testing platform utilizing hPSC-CMs to assess the cardiotoxicity and effectiveness of drugs. Following an optimized differentiation and maturation protocol, hPSC-CMs exhibited mature CM morphology, phenotype, and functionality, making them suitable for drug testing applications. We monitored intracellular calcium dynamics using calcium imaging techniques to measure spontaneous calcium oscillations in hPSC-CMs in the presence or absence of test compounds. For the cardiotoxicity test, hPSC-CMs were treated with various compounds, and calcium flux was measured to evaluate their effects on calcium dynamics. We found that cardiotoxic drugs withdrawn due to adverse drug reactions, including encainide, mibefradil, and cetirizine, exhibited toxicity in hPSC-CMs but not in HEK293-hERG cells. Additionally, in the effectiveness test, hPSC-CMs were exposed to ATX-II, a sodium current inducer for mimicking long QT syndrome type 3, followed by exposure to test compounds. The observed changes in calcium dynamics following drug exposure demonstrated the utility of hPSC-CMs as a versatile model system for assessing both cardiotoxicity and drug efficacy. Overall, our findings highlight the potential of hPSC-CMs in advancing drug discovery and development, which offer a physiologically relevant platform for the preclinical screening of novel therapeutics.

## 1. Introduction

Cardiovascular diseases (CVDs) continue to pose a significant global health challenge, necessitating the development of novel therapeutic strategies [1,2]. However, conventional drug discovery approaches often fall short of accurately predicting human cardiac responses, leading to high attrition rates in clinical trials. Therefore, there is an urgent need for more physiologically relevant preclinical models [3].

The emergence of human pluripotent stem cells (hPSCs) has revolutionized the field of regenerative medicine and drug discovery by providing a scalable and renewable source of human cardiac cells. These hPSCs, including both embryonic stem cells (ESCs) and induced pluripotent stem cells (iPSCs), have the ability to self-renew indefinitely and differentiate into virtually any cell type, including cardiomyocytes (CMs). Recent studies revealed that CMs derived from hPSCs (hPSC-CMs) could offer a unique opportunity to bridge the gap between traditional preclinical models and human physiology [4,5,6]. These hPSC-CMs exhibit high similarity to their native counterparts in terms of morphology, contractile function, and drug response profiles, which are crucial for drug screening and toxicity testing.

The utilization of hPSC-CMs holds significant promise for reducing reliance on animal models, minimizing the risk of adverse drug reactions in humans, and accelerating the pace of drug discovery and development [7,8]. By leveraging patient-specific iPSCs, researchers can also model genetic predispositions to CVDs and gain a better understanding of individual drug responses, paving the way for precision medicine approaches [9,10,11]. However, several challenges remain, including the need to enhance the maturity, scalability, and standardization of hPSC cm cultures.

This study aimed to provide a quick and pragmatic method to generate and utilize hPSC-CMs in drug screening and cardiotoxicity assessment. We also tried to establish a modified protocol for integrating hPSC-CMs with high-throughput assays to facilitate efficient and cost-effective drug screening. Our findings could have implications for harnessing the power of hPSC-CMs to accelerate the translation of basic science discoveries into clinically relevant therapies and ultimately improve patient outcomes in the treatment of CVDs.

## 2. Results

### 2.1. Optimization of Culture Conditions for hPSC cm Generation

**Figure 1:**
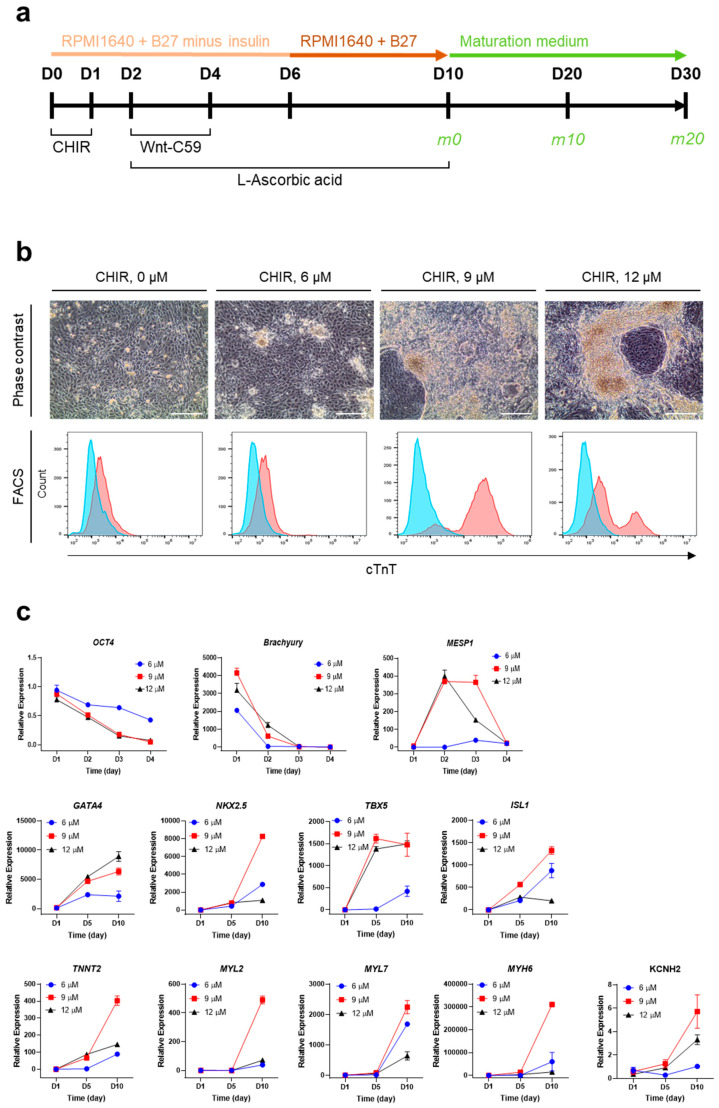
Optimization of culture conditions for the generation of hPSC-CMs. (**a**) Culture scheme for efficient differentiation and maturation of iPSCs. CHIR, CHIR99021. (**b**) Optimization of CHIR99021 concentration for inducing iPSC differentiation based on the expression of cTnT on D10. Upper, representative phase contrast images. Scale bar, 100 μm. Lower, representative FACS histograms. The blue area represents the isotype control, and the red area represents anti-cTnT antibody. (**c**) Relative mRNA levels of CM-related genes during the differentiation of iPSCs. mRNA levels of individual genes were normalized to undifferentiated cells (D0). Error bars, mean ± SEM (N = 3).

To optimize the culture conditions for CM generation from hPSCs (iPSCs and ESCs), cells were propagated on Matrigel-coated culture plates with maintenance medium, as described in the Section 4. Subsequently, hPSCs underwent differentiation for 10 days, followed by maturation for another 10 days or longer if needed (Figure 1a). For the initial induction of mesodermal differentiation, CHIR99021, a GSK3 inhibitor and Wnt activator, was used. On the first day of hPSC cm generation, hPSCs were treated with CHIR99021 at various concentrations (0, 6, 9, and 12 μM) (Figure 1b). The optimal concentration of CHIR99021 for the efficient generation of hPSC-CMs was determined to be 9 μM based on their morphology and cTnT expression (Figure 1b). For further differentiation into cardiac progenitor cells, Wnt-C59, a small molecule that inhibits Wnt palmitoylation, was added to the differentiation medium from D2 to D4. For late differentiation into contracting CMs from D6 to D10, insulin was added. Contracting CMs were observed between D9 and D11 (Supplementary Figure S1). During differentiation over 10 days, there were changes in the expression levels of CM-related genes (Figure 1c). In particular, *OCT4* (pluripotency) and *BRACHYURY* (mesodermal specification) were gradually decreased in the first 4 days, and *MESP1* (early cardiovascular development) was initially increased on D2 and sharply decreased on D4. Moreover, *GATA4*, *NKX2.5*, *TBX5*, and *ISL1*, transcription factors that play key roles in cardiac development [12,13,14], were highly expressed on D10 compared with D0 or D5, indicating the induction of cardiac lineage cells. In addition, *TNNT2*, *MYL2*, *MYL7*, *MYH6*, and *KCNH2*, genes encoding proteins serving as markers for mature and functional CMs, were markedly increased on D10 compared with D5. Based on the results, our culture conditions were optimal for hPSCs to give rise to cells highly expressing CM-specific genes.

### 2.2. Culture Supplementation with FFA to Accelerate CM Maturation

**Figure 2:**
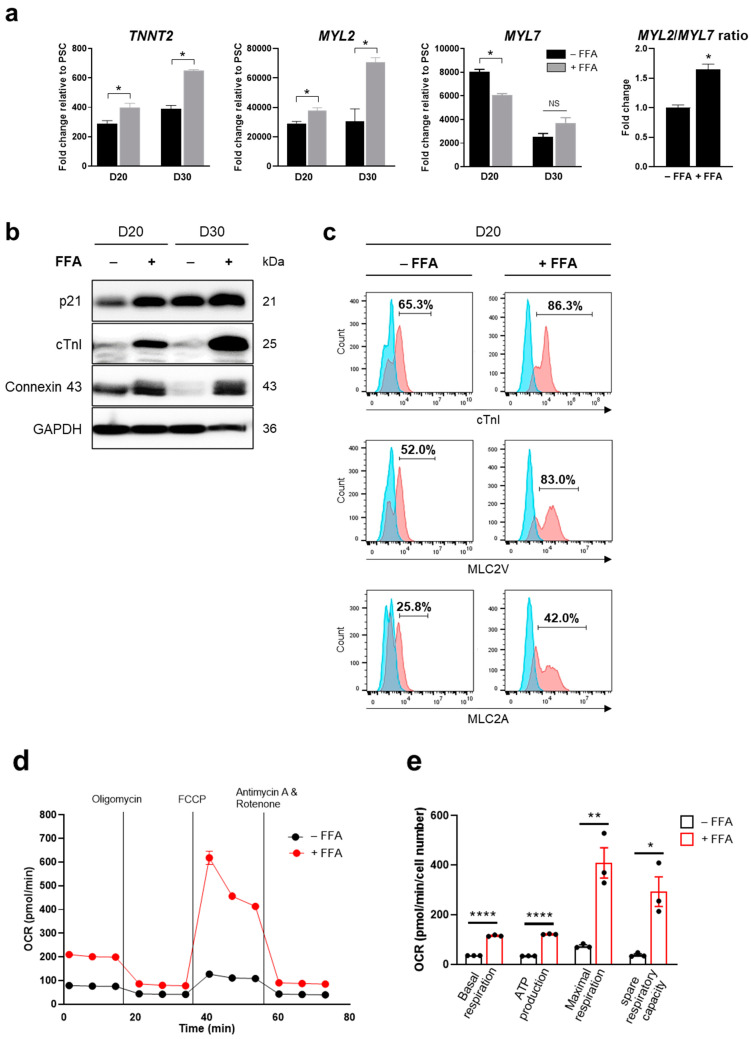
FFA-accelerated CM differentiation from hPSCs. (**a**,**b**) Expression levels of CM markers in iPSC-CMs on D20 and D30. The relative mRNA levels of *TNNT2*, *MYL2*, and *MYL7* were quantified by qPCR and the *MYL2*/*MYL7* ratio on D20 was calculated (**a**). Protein expression levels of p21, cTnI, and Connexin 43 were measured by Western blotting (**b**). (**c**) Cell populations expressing CM markers on D20. Cells positive for cTnI, MLC2V, and MLC2A were increased in the presence of FFA. The blue area represents the isotype control, and the red area represents the specific antibody. (**d**,**e**) Measurement of OCR as an indicator of cellular oxygen consumption and mitochondrial respiration. The OCR of FFA-treated iPSC-CMs was elevated on D20. Error bars, mean ± SEM (N = 3). Asterisks, *, significant differences (Student’s *t*-test; *, *p* < 0.05; **, *p* < 0.01; ****, *p* < 0.001). NS, no statistical significance.

To accelerate CM maturation, a free fatty acid mixture (FFA) was added to the culture starting from D10. We observed that the addition of FFA to the medium increased the expression levels of mature CM markers, including *TNNT2*, *MYL2*, and *MYL7* at the mRNA level (Figure 2a) and p21 (a cyclin-dependent kinase inhibitor), cTnI, and Connexin 43 (a gap junction protein) at the protein level (Figure 2b; Supplementary Figure S2). Connexin 43, critical for coordinating cardiac contraction, was immunostained in differentiated hPSC-CMs (Supplementary Figure S3). Sarcomeric bands were also observed with immunostaining using antibodies against cTnI and cTnT (Supplementary Figure S3). In addition, compared with cells cultured without FFA, those cultured in FFA-containing maturation medium for 10 days (on D20) expressed higher levels of cTnI (a regulatory protein of the troponin complex in cardiac muscle), MLC2V (a myosin light chain protein predominantly expressed in ventricular cardiac muscle), and MLC2A (a myosin light chain protein predominantly expressed in atrial cardiac muscle) (Figure 2c). This indicated that FFA supplementation facilitated the expression of genetic and phenotypic characteristics of mature CMs. Furthermore, the OCR, indicative of basal respiration, ATP production, maximal respiration, and spare respiratory capacity, was significantly elevated in FFA-treated iPSC-CMs (Figure 2d,e) and ESC-CMs (Supplementary Figure S4a,b) on D20. These observations were consistent with the metabolic feature primarily relying on oxidative phosphorylation for energy production in matured hPSC-CMs.

### 2.3. Determination of hPSC cm Functionality Based on Electrophysiological Properties

**Figure 3:**
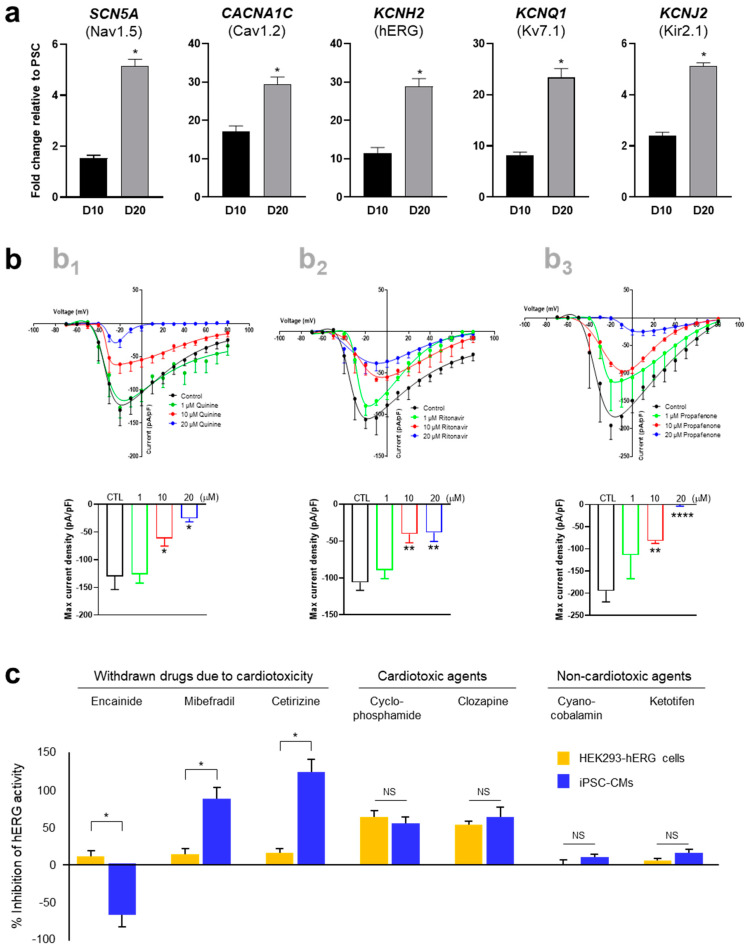
Functional maturation of hPSC-CMs. (**a**) Relative mRNA levels of ion channels in iPSC-CMs. The mRNA levels of genes expressing Nav1.5, Cav1.2, hERG, Kv7.1, and Kir2.1 in iPSC-CMs were compared with those in undifferentiated iPSCs on D10 and D20. (**b**) Electrophysiological properties of iPSC-CMs in the presence of voltage-sensitive sodium channel blockers. Cells on D20 were treated with quinine (**b_1_**), ritonavir (**b_2_**), and propafenone (**b_3_**). (**c**) Electrophysiological activity of hERG channels in iPSC-CMs on D20 compared with HEK293-hERG cells. Cardiotoxic drugs withdrawn due to adverse drug reactions, including encainide, mibefradil, and cetirizine at 5 μM, exhibited toxicity in iPSC-CMs but not in HEK293-hERG cells. Error bars, mean ± SEM (N = 3). Asterisks, *—statistically significant differences (Student’s *t*-test; *p* < 0.05). NS—no statistical significance.

The maturity and functionality of hPSC-CMs were assessed based on ion channel expression and electrophysiological properties. In comparison with iPSC-CMs cultured for 10 days (D10), iPSC-CMs cultured in FFA-containing maturation medium for another 10 days (D20) showed high expression of ion channels, including Nav1.5, Cav1.2, hERG, Kv7.1, and Kir2.1 (Figure 3a). The electrophysiological activity of iPSC-CMs was examined by single-cell patch-clamping to measure ion channel currents. Specifically, voltage-sensitive sodium channels in iPSC-CMs were detected to evaluate excitation–contraction coupling using sodium channel-specific blockers, including quinine, ritonavir, and propafenone (Figure 3b). To further assess the potential of hPSC-CMs as a drug testing model system, the hERG channel activity of iPSC-CMs was evaluated and compared with that of HEK293-hERG cells in the presence of various drugs at a concentration of at 5 μM (Figure 3c). Cardiotoxic drugs withdrawn due to adverse drug reactions, including encainide, mibefradil, and cetirizine, exhibited toxicity in iPSC-CMs but not in HEK293-hERG cells. Non-cardiotoxic drugs showed similar results between iPSC-CMs and HEK293-hERG cells. The results indicated that iPSC-CMs differentiated under our culture conditions exhibited CM morphology, phenotype, and functionality, which may serve as a physiologically relevant model for detecting cardiotoxicity and testing the effectiveness of potential medications.

### 2.4. hPSC-CMs as a High-Throughput Screening Platform for Cardiotoxicity and Drug Efficacy

**Figure 4:**
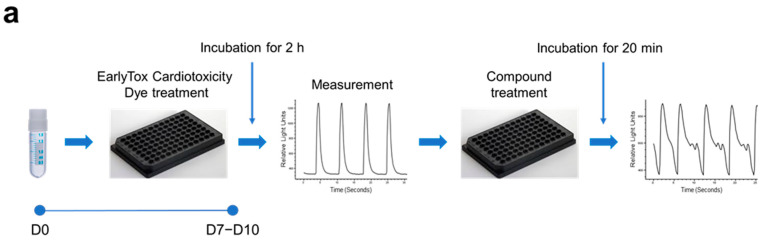

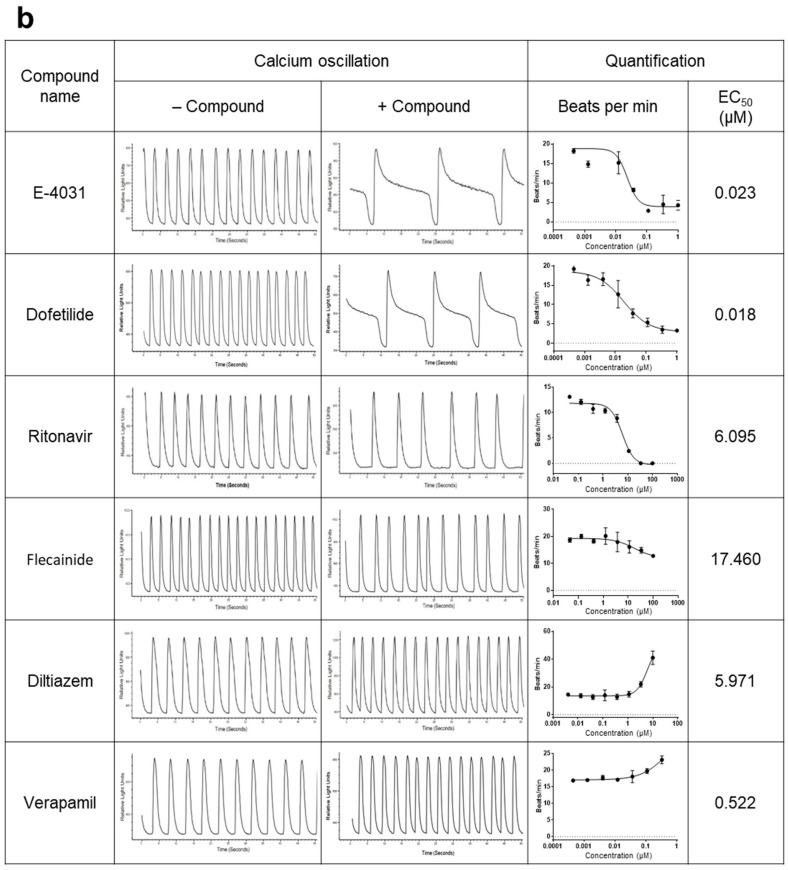

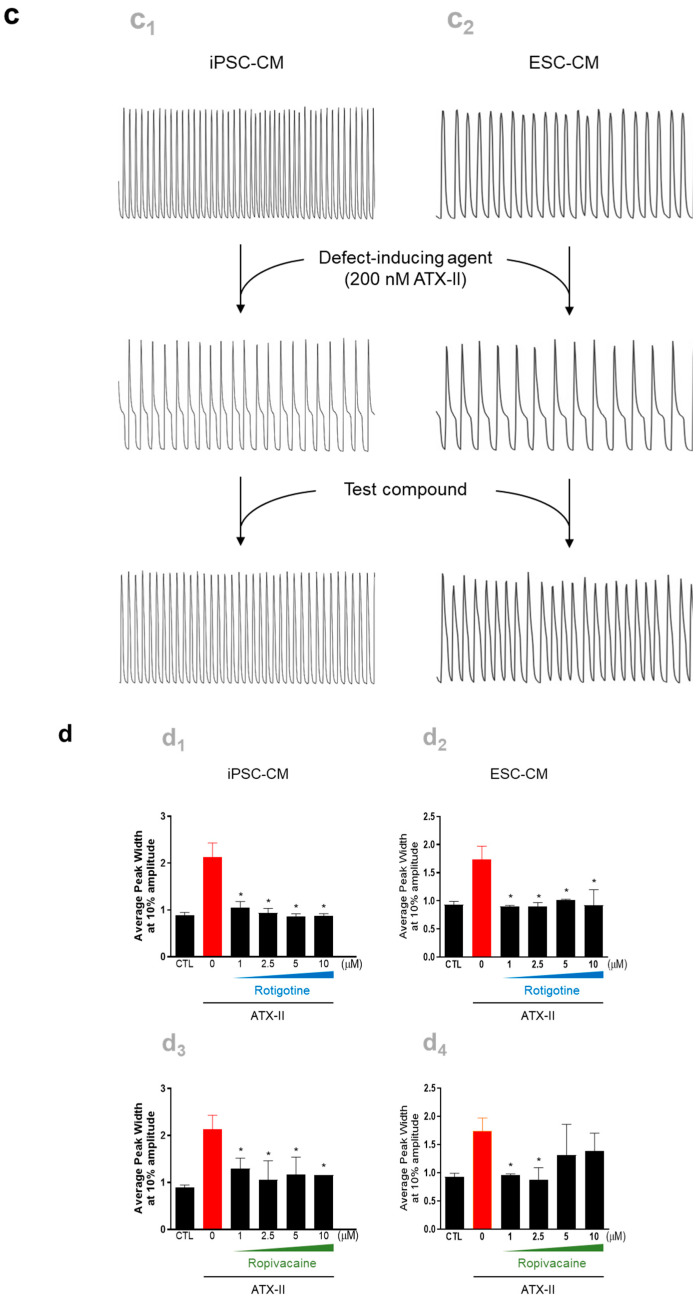
Utilization of hPSC-CMs as a platform for high-throughput testing of the cardiotoxicity and effectiveness of drugs. Spontaneous calcium oscillations in iPSC-CMs were measured using the FLIPR system in the absence or presence of test compounds. (**a**,**b**) Cardiotoxicity test. (**a**) Experimental scheme. iPSC-CMs loaded with a calcium-sensitive fluorescent dye, EarlyTox^TM^, were treated with a test compound, and calcium flux was observed. (**b**) Testing of the compounds known for their adverse effects in humans. The dye-loaded cells were exposed to ion channel blockers targeting hERG channels (E-4031 and dofetilide), sodium channels (ritonavir and flecaninide), and L-type calcium channels (diltiazem and verapamil) at designated concentrations, ranging from the highest concentration to serially diluted 1:3, while calcium oscillations were recorded. (**c**,**d**) Efficacy test. (**c**) iPSC-CMs (**c_1_**) and ESC-CMs (**c_2_**) were treated with 200 nM ATX-II, a late inward sodium current inducer, which produces atrial arrhythmias mimicking the LQT3 phenotype, followed by exposure to a test compound. (**d**) The elevated average peak widths of both iPSC-CMs (**d_1_**,**d_3_**) and ESC-CMs (**d_2_**,**d_4_**) by ATX-II treatment were restored to the level of untreated control cells after exposure to rotigotine (**d_1_**,**d_2_**) or ropivacaine (**d_3_**,**d_4_**). Error bars, mean ± SEM (N = 3). Asterisks, *—statistically significant differences (Student’s *t*-test; *p* < 0.05).

Using our iPSC-CMs, we developed a high-throughput testing platform for assessing the cardiotoxicity and effectiveness of drugs. Given that the iPSC-CMs in this study exhibited the maturity and functionality of CMs, we monitored intracellular calcium dynamics using fluorescence calcium imaging techniques. Spontaneous calcium oscillations in iPSC-CMs were measured using the FLIPR system in the absence or presence of test compounds. For the cardiotoxicity test (Figure 4a), iPSC-CMs loaded with a calcium-sensitive fluorescent dye were treated with test compounds, and calcium flux was observed. The test compounds used in this study were ion channel blockers targeting hERG channels (E-4031 and dofetilide), sodium channels (ritonavir and flecaninide), and L-type calcium channels (diltiazem and verapamil) (Figure 4b). The beating rate was altered depending on the concentration of each blocker, consistent with their respective modes of action. hERG channel blockers, E-4031 and dofetilide, caused a decrease in the beating rate and concentration-dependent prolongation of peak intervals (EC_50_ of 0.023 μM and 0.018 μM, respectively). Ritonavir and flecainide, sodium channel blockers, inhibited the beating rates in a concentration-dependent manner (EC_50_ of 6.095 μM and 17.460 μM, respectively). Diltiazem or verapamil, calcium channel blockers, increased the beating rate (EC_50_ of 5.971 μM and 0.522 μM, respectively). This approach allowed the evaluation of proarrhythmic effects of drug candidates based on calcium dynamics occurring within the iPSC-CMs. 

For the efficacy test (Figure 4c), iPSC-CMs and ESC-CMs were generated as previously mentioned and used to screen effective compounds for reversing arrhythmia phenotype. The CMs were exposed to 200 nM ATX-II, a late inward sodium current inducer known to produce atrial arrhythmias, to mimic the contraction-associated spontaneous calcium oscillations of long QT syndrome type 3 (LQT3), followed by exposure to test compounds. Following exposure to ATX-II, the higher average peak widths were observed in both iPSC-CMs and ESC-CMs (Figure 4d). Among the five test compounds, including estradiol, nicotinamide, clobetasol, ropivacaine, and rotigotine, rotigotine and ropivacaine significantly reverted the average peak width to the level of untreated control cells (Figure 4d). These findings demonstrated the utility of hPSC-CMs as a model system for assessing both the cardiotoxicity and effectiveness of potential therapeutic compounds.

## 3. Discussion

CMs derived from human stem cells have emerged as a promising tool for studying cardiac physiology and drug responses. However, their usage is mainly limited by a structurally and functionally immature phenotype and lack of high-throughput analytical methodology. The optimization of culture conditions for the generation of functional CMs from hPSCs, including iPSCs and ESCs, is a critical step for their effective utilization in drug screening assays aimed at assessing both cardiotoxicity and therapeutic efficacy in various disease contexts [6,15,16]. 

In this study, we developed a high-throughput testing platform utilizing hPSC-CMs to assess the cardiotoxicity and effectiveness of drugs. Our protocol, involving the modulation of the differentiation process and supplementation with FFA, could enhance the phenotypic and functional characteristics of hPSC-CMs, making them suitable for drug testing applications.

The hPSC-CMs generated and matured under our optimized culture conditions were found to recapitulate key aspects of cardiac development and maturation in vitro, thereby closely resembling their native counterparts [7,17,18,19]. By culturing hPSCs on Matrigel-coated plates and inducing mesodermal differentiation using CHIR99021, a Wnt activator [20,21,22], we successfully initiated cardiac differentiation. An optimal concentration of CHIR99021 was used based on morphological characteristics and cTnT expression, which underscores the importance of the precise modulation of signaling pathways in driving efficient CM differentiation. Subsequent supplementation with Wnt-C59 and insulin further induced the differentiation and maturation of CMs, leading to the emergence of contracting CMs with enhanced expression of mature cardiac markers [23,24,25]. 

Given that early-stage CMs primarily use glucose as their energy source, whereas mature CMs rely on fatty acid-based oxidative phosphorylation to generate ATP [26,27,28], we leveraged the metabolic shift that occurs during CM development [29,30]. To expedite the maturation, differentiated hPSC-CMs were cultured in maturation medium containing FFA and no glucose for another 10 days. Notably, supplementation with FFA accelerated CM maturation, as demonstrated by the increased expression of CM markers and elevated OCR, indicating improved mitochondrial respiration and metabolic activity [26,31,32,33]. These findings highlight the dynamic interplay between supplementing substances in culture media and cellular metabolic responses, underscoring the importance of optimizing culture parameters for the generation of functional CMs.

In the context of drug screening, calcium imaging techniques could facilitate the assessment of cardiotoxicity by monitoring intracellular calcium dynamics in hPSC-CMs [6,34,35,36]. Spontaneous calcium oscillations were observed under basal conditions and in the presence of various test compounds, offering insights into their effects on calcium handling and potential cardiotoxic effects [37,38,39].

Taken together, the findings suggest that hPSC-CMs may serve as a physiologically relevant model system for assessing both cardiotoxicity and therapeutic efficacy [6,40]. By exposing hPSC-CMs to candidate compounds, researchers can evaluate their effects on various aspects of cardiac function, including electrophysiological properties, contractility, and viability [41,42,43]. High-throughput screening methods, such as multi-electrode array recordings and fluorescence-based assays, allow the systematic evaluation of large compound libraries, thus highlighting potential adverse effects and therapeutic benefits [44,45].

Furthermore, patient-specific hPSC-CMs offer a unique opportunity for personalized medicine approaches, which can identify drugs that are the most effective for individual patients based on their genetic background and disease characteristics [7,46,47]. Incorporating disease-specific features into hPSC cm models allows the simulation of drug-induced cardiotoxicity associated with certain diseases or comorbidities, providing valuable insights into underlying mechanisms and strategies to mitigate adverse effects [48].

In conclusion, the optimization of culture conditions for hPSC cm generation and their subsequent utilization in drug screening assays represent a feasible approach to advance our understanding of cardiac biology, improve the safety and efficacy of pharmaceutical interventions, and ultimately enhance patient outcomes in the treatment of CVDs and beyond.

## 4. Materials and Methods

### 4.1. Maintenance of hPSCs

The iPSC (ATCC^®^ ACS-1011™) and ESC (WA09, referred to as H9) cell lines were obtained from ATCC (Manassas, VA, USA) and WiCell Research Institute (Madison, WI, USA), respectively. The cells were plated on culture plates coated with Matrigel^®^ Growth Factor reduced Basement Membrane Matrix (Corning Life Sciences, Corning, NY, USA) at a dilution of 1:30 in maintenance medium. The maintenance medium for hPSC cultures was Pluripotent Stem Cell SFM XF/FF (ATCC) or mTeSR™1 (STEMCELL Technologies, Vancouver, BC, Canada). Both hPSC cell lines were passaged at a 1:6 ratio when the confluency reached around 80% using Dispase (STEMCELL Technologies). The experimental data were obtained using CMs differentiated from hPSCs, including iPSCs and ESCs, according to previously published protocols [21,49] with modifications as described below.

### 4.2. Generation of hPSC-CMs

For differentiation into CMs, hPSCs were seeded on Matrigel^®^-coated plates and cultured with maintenance medium in the presence of 10 μM Y27632 (Selleck Chemicals, Houston, TX, USA). After 24 h, the medium was replenished every day for 3 days until the cells achieved a confluency above 90%. Subsequently, the cells were cultivated in differentiation medium for 10 days (Figure 1a). The initial differentiation medium consisted of RPMI1640 (Gibco/Thermo Fisher Scientific, Waltham, MA, USA) supplemented with B27^TM^ Minus Insulin (Gibco/Thermo Fisher Scientific) for 6 days (from D0 to D6). On D0, CHIR99021 (Selleck Chemicals, Houston, TX, USA) was added to the culture, and it was removed from the medium on D1. On D2, the medium was replaced with the initial differentiation medium containing 2 μM Wnt-C59 (Selleck Chemicals) and 50 μg/mL L-ascorbic acid (Sigma-Aldrich, St. Louis, MO, USA). On D4, the medium was refreshed with the initial differentiation medium containing only L-ascorbic acid. From D6, the medium was refreshed with RPMI1640 supplemented with B27 and ascorbic acid every other day. CM contraction was generally observed from D8 to D11.

### 4.3. Maturation of hPSC-CMs

For maturation, differentiated hPSC-CMs were cultured in maturation medium for another 10–20 days. The maturation medium was composed of RPMI1640 (no glucose) supplemented with B27, 10 mM galactose, 1 mM L-glutamine, 50 μg/mL ascorbic acid, 10 nM triiodothyronine (Sigma-Aldrich), 50 μM palmitic acid (Sigma-Aldrich), and 1× Linoleic Acid–Oleic Acid–Albumin (Sigma-Aldrich). The medium was replenished every other day.

### 4.4. Real-Time Quantitative PCR (qPCR) Analysis

Total RNA was extracted from the harvested cells using TRIzol^TM^ Reagent (Thermo Fisher Scientific). The extracted RNA (1 μg) was reverse-transcribed into first-strand cDNA using the High-Capacity cDNA Reverse Transcription Kit (Applied Biosystems, Waltham, MA, USA) according to the manufacturer’s instructions. qPCR was performed with the PowerUp^TM^ SYBR^TM^ Green Master Mix (Applied Biosystems) and StepOnePlus^TM^ Real-Time PCR System (Applied Biosystems). The mRNA quantity of the gene of interest was expressed relative to the level of a housekeeping gene (GAPDH). The calculation was performed using the ΔΔCT method based on the threshold cycle (CT) as 2^−Δ(ΔCT)^, where ΔCT = CT_gene of interest_ − CT_GAPDH_ and Δ(ΔCT) = ΔCT_differnetiated_ − ΔCT_control_. The primer sequences are shown in Supplementary Table S1.

### 4.5. Flow Cytometry

Differentiated hPSC-CMs were dissociated with trypsin/EDTA, and 5 × 10^5^ cells were harvested. After fixation, the cells were permeabilized with the Cytofix/Cytoperm Solution Kit (BD Biosciences, Franklin Lakes, NJ, USA) and immunostained with primary antibodies, including mouse anti-cardiac muscle troponin T (cTnT; 1:100; Abcam, Cambridge, UK), rabbit anti-cardiac muscle troponin I (cTnI; 1:100; Abcam), rabbit anti-myosin light chain 2v (MLC2V; 1:50; ProteinTech, Rosemont, IL, USA), and mouse anti-myosin light chain 2a (MLC2A; 1:50; Synaptic Systems, Göttingen, Germany). After rinsing with Perm/Wash Buffer (BD Biosciences), appropriate secondary antibodies were introduced: Alexa-488-conjugated goat anti-mouse IgG, Alexa-488-conjugated goat anti-rabbit IgG, and Alexa-647-conjugated goat anti-mouse IgG (Molecular Probes, Eugene, OR, USA) at a dilution of 1:200. The stained cells were then sorted using FACS Aria III (Becton Dickinson, Franklin Lakes, NJ, USA) based on forward scatter, side scatter, and fluorescence intensity parameters adjusted to the recommended excitation and emission wavelengths. At least 10,000 gated cells were acquired and analyzed, and histogram plots were generated using FlowJo software v10.10 (BD Biosciences). 

### 4.6. Western Blot Analysis

Cells were harvested and lysed in lysis buffer (40 mM Tris-HCl, pH 8.0, 120 mM NaCl, 0.1% Nonidet P-40) supplemented with a protease inhibitor cocktail (Roche, Mannheim, Germany), followed by centrifugation. Clarified cell lysates were loaded onto SDS-PAGE gels at a quantity of 20 μg per well. After electrophoresis, proteins were transferred onto a nitrocellulose membrane. After soaking in blocking buffer (5% *w*/*v* skim milk in Tris-buffered saline, pH 7.4, including 0.1% *v*/*v* Tween 20) for 1 h, the membrane was incubated with primary antibodies overnight at 4 °C, followed by secondary antibodies for 1 h at room temperature. The antibodies were IgGs against p21 (Sigma-Aldrich), cTnI (Abcam), Connexin 43 (Cell Signaling Technology, Beverly, MA, USA), and GAPDH (Santa Cruz Biotechnology, Dallas, TX, USA) at a dilution of 1:1000. Antibody binding was visualized using horseradish peroxidase-conjugated goat anti-rabbit or anti-mouse secondary antibodies at a dilution of 1:5000 and an enhanced chemiluminescence system (GE Healthcare, Buckinghamshire, UK). Protein bands were detected and digitalized using LuminoGraph III Lite (ATTO, Tokyo, Japan).

### 4.7. Immunocytochemistry

Differentiated hPSC-CMs were dissociated with Trypsin/EDTA, plated onto Matrigel^®^-coated glass coverslips, and cultured for a designated period. The cells were then fixed with 4% paraformaldehyde solution (Sigma-Aldrich), rinsed with phosphate-buffered saline (PBS), and blocked with 5% normal serum and 0.2% Triton X-100 in PBS. The preparations were allowed to react with primary antibodies overnight at 4 °C. The primary antibodies used in this study were as follows: mouse anti-cTnT (1:100; Abcam), rabbit anti-cTnI (1:100; Abcam), and rabbit anti-Connexin 43 (1:100; Cell Signaling Technology). On the next day, the cells were rinsed with PBS and incubated with Alexa-488-conjugated goat anti-mouse or anti-rabbit and Alexa-568-conjugated donkey anti-rabbit IgG (1:200; Molecular Probes) for 1 h at room temperature. Cell nuclei were counterstained with DAPI (1:2500; Molecular Probes). Images were observed and visualized using a fluorescence microscope (Eclipse Ti-U; Nikon, Tokyo, Japan). 

### 4.8. Measurement of Oxygen Consumption Rate (OCR) of hPSC-CMs

Mitochondrial respiration was evaluated using the Seahorse XFe96 Extracellular Flux Analyzer (Agilent Technologies, Santa Clara, CA, USA) according to the manufacturer’s protocols. Seahorse cell culture microplates were coated with diluted Matrigel^®^. The prepared hPSC-CMs were seeded on the plates at a density of 3 × 10^4^ cells per well and cultured in the appropriate culture medium for 2 days in a humidified incubator (5% CO_2_ and 37 °C) before being assayed. The culture medium was changed to Agilent Seahorse XF DMEM Basal Medium supplemented with 2 mM glutamine, 10 mM glucose, and 1 mM sodium pyruvate 1 h before the assay and for the duration of the measurement. The Mito Stress Test was performed using 1 μM oligomycin, 1 μM FCCP, and a 0.5 μM mixture of rotenone and antimycin A. The OCR was normalized to the number of cells captured in a field of view (10×) for each well quantified by DAPI staining. 

### 4.9. Whole-Cell Patch-Clamp Recording

For whole-cell patch-clamping, glass coverslips were placed into each well of a 4- or 24-well plate and coated with diluted Matrigel. hPSC-CMs were seeded on each coverslip at a density of 5 × 10^4^ cells per well, which were maintained as single cells and cultured in maturation medium for 10 days (until ‘D20’). The medium was refreshed every other day before use. hPSC-CMs grown on the coverslips were transferred to a recording chamber where an external solution was circulated at 2 mL/min for recording. Nav1.5 sodium channel currents were measured by incrementally increasing the voltage by 10 mV from a holding potential of -70 mV to +80 mV. The pipette solution composition was as follows: 130 mM CsCl, 5 mM NaCl, 5 mM tetraethylammonium (TEA)-Cl, 2.5 mM HEPES, and 1 mM ethylene glycol-bis(2-aminoethyl ether)-N,N,N′,N′-tetraacetic acid (EGTA), adjusted to pH 7.2 with CsOH. The extracellular bath solution used for recording sodium ion channel activity in cells comprised 140 mM NaCl, 10 mM HEPES, 1 mM CaCl_2_, 1.4 mM MgCl_2_, 5 mM KCl, and 10 mM glucose, adjusted to pH 7.4 with NaOH. Quinine (Sigma-Aldrich), ritonavir (Sigma-Aldrich), and propagenone (Sigma-Aldrich) were used as antagonists.

For measurement of human ether-à-go-go-related gene (hERG) current, a hERG-overexpressing HEK293 cell line (provided from Eurofins Scientifics, Luxembourg) was utilized. These cells were cultured on coverslips in Dulbecco’s Modified Eagle Medium/Nutrient Mixture F-12 (DMEM/F-12) supplemented with 1× GlutaMAX™, 10% fetal bovine serum (Merck Co., Rahway, NJ, USA), 400 µg/mL of G418 (Gibco/Thermo Fisher Scientific) and 1% non-essential amino acids. HEK293-hERG cells, prepared on coverslip substrates, were transferred to a recording chamber where an external solution was continuously circulated at a rate of 2 mL/min. The external solution composition consisted of 140 mM NaCl, 5.4 mM KCl, 1.8 mM CaCl_2_, 1 mM MgCl_2_, 10 mM HEPES, and 10 mM glucose, adjusted to pH 7.4 with HCl. The pipette solution composition was as follows: 120 mM KCl, 1 mM MgCl_2_, 3 mM Mg-ATP, 10 mM HEPES, and 10 mM EGTA, adjusted to pH 7.2 with KOH. A micro glass electrode, with an impedance ranging from 3 to 5 MΩ and containing an internal solution, was employed to establish whole-cell patch configurations. The access resistance (Ra) of the whole-cell patch clamp was maintained within the range of 10 to 20 MΩ. Membrane potential was systematically modulated in a stepwise manner: −70 mV for 2 s, −50 mV for 0.5 s, −70 mV for 0.5 s, +40 mV for 2 s, −50 mV for 2 s, and −70 mV for 2 s. Peak currents mediated by hERG channel activation were quantified as the average value obtained from five consecutive measurements. Subsequently, the same cells were exposed to the test substance at varying concentrations, and after an incubation period of 5 min, the same voltage protocol was used to measure peak hERG channel currents. The test substances were as follows: encainide, mibefradil, cetirizine, cyclophosphamide, clozapine, cyanocobalamin, and ketotifen (all from Sigma-Aldrich). hERG currents were recorded using the Axopatch 700B amplifier and DigiData 1440A digitizer in the voltage clamp mode. Data analysis was performed using pCLAMP software (version 10.4; Molecular Devices, San Jose, CA, USA).

### 4.10. Measurement of Calcium Flux 

Spontaneous calcium transients in contracting CMs were monitored using the FLIPR Tetra^®^ High Throughput Cellular Screening System (Molecular Devices). hPSC-CMs were plated on the Matrigel-coated wells (8 × 10^4^ cells per well) of a 96-well plate. Confluency was monitored after 2 days, and the strong synchronous contractions of cells were confirmed prior to the cardiac calcium oscillation assay. Experiments were conducted after 7–10 days of cell culture. The EarlyTox^TM^ Cardiotoxicity Kit (Molecular Devices) was used; a calcium-sensitive fluorescent dye was added to the plate according to the manufacturer’s instructions and incubated for 2 h at 37 °C. Before treatment with test compounds, basal calcium oscillations were recorded at an excitation wavelength of 470–495 nm and an emission wavelength of 515–575 nm for 130 s with a 0.125 s read interval. The test compounds, E-4031 (Sigma-Aldrich), dofetilide (Tocris, Bristol, UK), ritonavir (Sigma-Aldrich), flecainide (Sigma-Aldrich), diltiazem (Tocris), and verapamil (Tocris), were, respectively, added to the wells of the culture plates at the designated concentrations. Calcium oscillations were measured at 20, 40, and 60 min after treatment. The acquired data were analyzed for beat frequency and peak width at 10% amplitude using ScreenWorks Peak Pro software version 2.0 (Molecular Devices).

### 4.11. Statistical Analysis

Values are expressed as the mean ± standard error of the mean (SEM) of three independent experiments unless otherwise indicated. The two groups were evaluated by Student’s *t*-test, and multiple comparisons between data were analyzed with one-way analysis of variance (ANOVA). Statistical analyses were conducted using GraphPad Prism 8 software (GraphPad Software Inc., La Jolla, CA, USA), and a *p*-value of <0.05 was considered statistically significant.

## Data Availability

The authors declare that all data supporting the findings of this study are available in the article and can be provided by the corresponding author upon reasonable request.

## Supplementary Materials

The supporting information can be downloaded at: https://www.mdpi.com/article/10.3390/ijms25147971/s1.
