# Supplementary material for: Advancing Cardiovascular Drug Screening Using Human Pluripotent Stem Cell-Derived Cardiomyocytes
Source: Int J Mol Sci. 2024 Jul 21;25(14):7971. doi: 10.3390/ijms25147971 (PMC11277421; doi:10.3390/ijms25147971)
Supplement: Supplementary file 1 [file ijms-25-07971-s001.zip › ijms-3081928_Supplementary information_revised_re-organized.pdf]

Supplementary information

Supplementary Table

Supplementary Table S1. Primer sequences for qPCR

Supplementary Figures

Supplementary Figure S1. Generation of contracting iPSC-CMs.

Supplementary Figure S2. Western blot images for the graphs in Figure 2b.

Supplementary Figure S3. Differentiated iPSC-CMs on D20.

Supplementary Figure S4. Mitochondrial respiration in ESC-CMs.

Supplementary Table

Supplementary Table S1. Primer sequences for qPCR

Gene	Forward primer (5′→3′)	Reverse primer (5′→3′)
OCT4	GGGTTCTATTTGGGAAGGTAT	TTCATTGTTGTCAGCTTCCT
BRACHYURY	AAGAAGGAAATGCAGCCTCA	TACTGCAGGTGTGAGCAAGG
MESP1	TCGAAGTGGTTCCTTGGCAGAC	CCTCCTGCTTGCCTCAAAGTGTC
GATA4	ACACCCCAATCTCGATATGTTTG	GTTGCACAGATAGTGACCCGT
NXK2.5	ACCCTGAGTCCCCTGGATTT	TCACTCATTGCACGCTGCAT
TBX5	GAACCACAAGATCACGCAATTA	ACACCATTCTCACACTGGTAT
ISL1	ATTTCCCTATGTGTTGGTTGC	CGTTCTTGCTGAAGCCGATG
TNNT2	TTCACCAAAGATCTGCTCCTCGCT	TTATTACTGGTGTGGAGTGGGTGTGG
MYL2	CGCCAACTCCAACGTGTTCT	CCATCCCTGTTCTGGTCCAT
MYL7	CCCATCAACTTCACCGTCTTCCT	AGAGAACTTGTCTGCCTGGGTCA
MYH6	GACTGTTGTGGCCCTGTACC	GGAAGGATGAGCCCTTTTTC
SCN5A	AGCTGGCTGATGTGATGGTC	CACTTGTGCCTTAGGTTGCC
CACNA1C	ATGAACATGCCTCTGAACAGCG	TTCAGAAGCAGCGGACACAGC
KCNH2	TTCGACCTGCTCATCTTCGG	CGATGCGTGAGTCCATGTGT
KCNQ1	CGCCTGAACCGAGTAGAAGA	TGAAGCATGTCGGTGATGAG
KCNJ2	ACCGCTACAGCATCGTCTCT	TCCACACACGTGGTGAAGAT
GAPDH	ATGGAAATCCCATCACCATCTT	CGCCCCACTTGATTTTGG

Supplementary Figures

A video file (MP4) separately provided

Supplementary Figure S1. Generation of contracting iPSC-CMs. Spontaneous and regular contraction of monolayer cells over time.

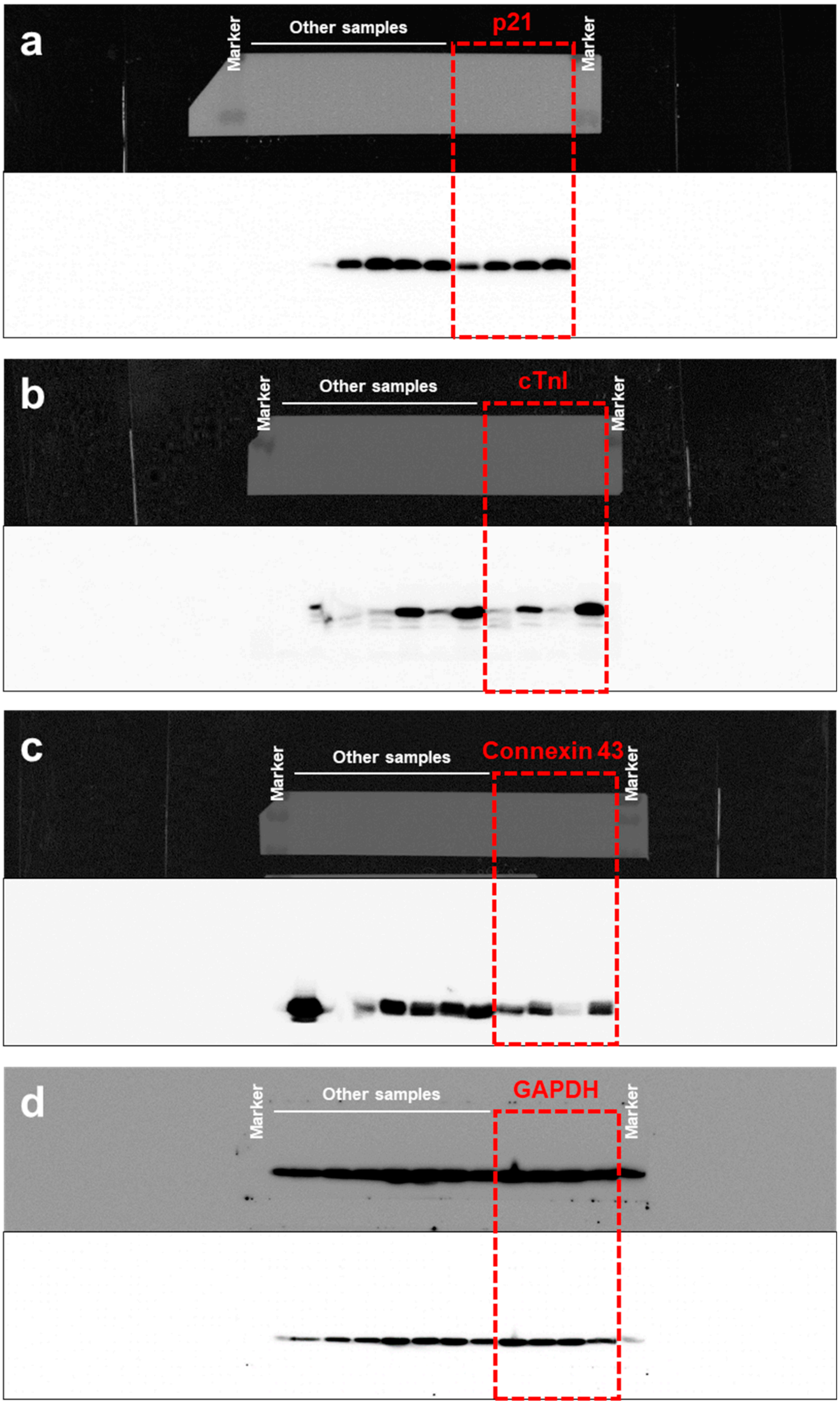

Supplementary Figure S2. Original Western blot images for Figure 2b. The protein expression levels of p21 (a), cTnI (b), and Connexin 43 (c) relative to GAPDH (d) were assessed by Western blotting.

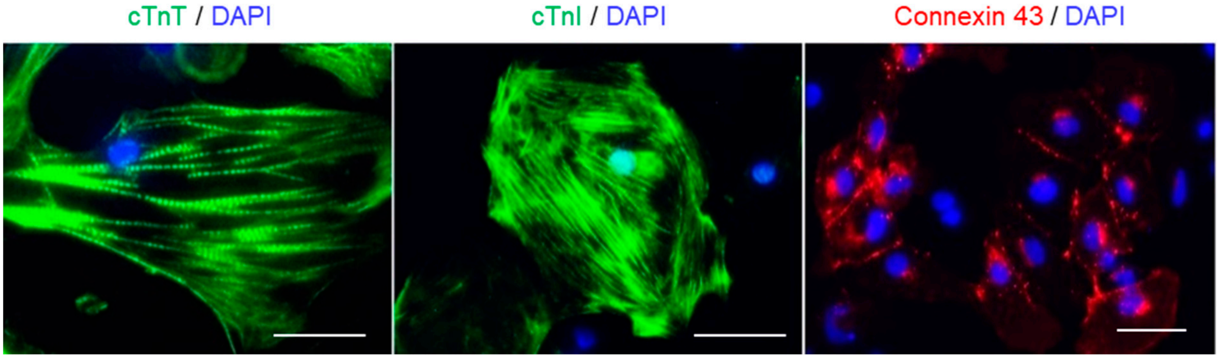

Supplementary Figure S3. Differentiated iPSC-CMs on D20. Differentiated iPSC-CMs were immunostained for cTnT, cTnI, and Connexin 43. Scale bar, 50 µm.

a

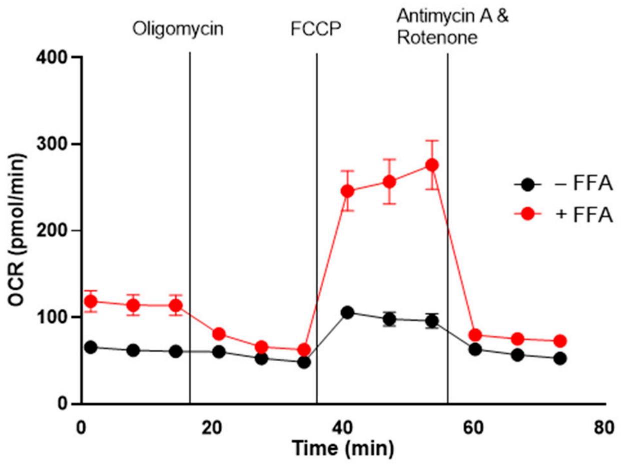

b

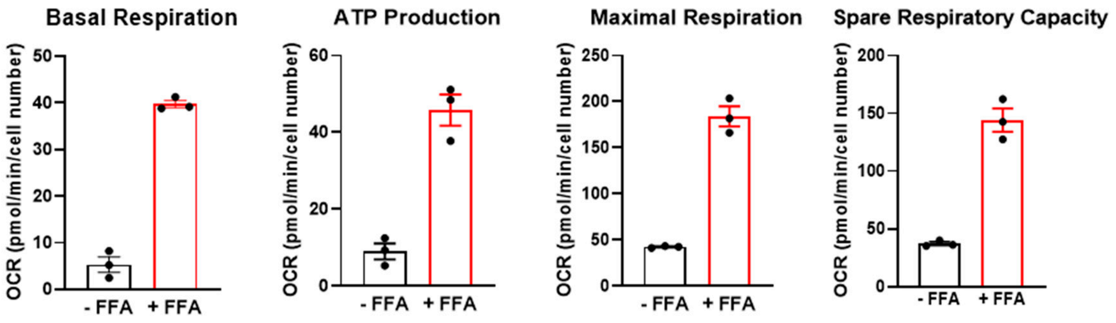

Supplementary Figure S4. Mitochondrial respiration in ESC-CMs. (a–b) Measurement of OCR as an indicator of cellular oxygen consumption and mitochondrial respiration. (a) The OCR of FFA-treated ESC-CMs was elevated on D20 compared with untreated cells. (b) Basal respiration, ATP production, maximal respiration, and spare respiratory capacity in both CMs cultured in the absence and presence of FFA supplementation were quantified.
